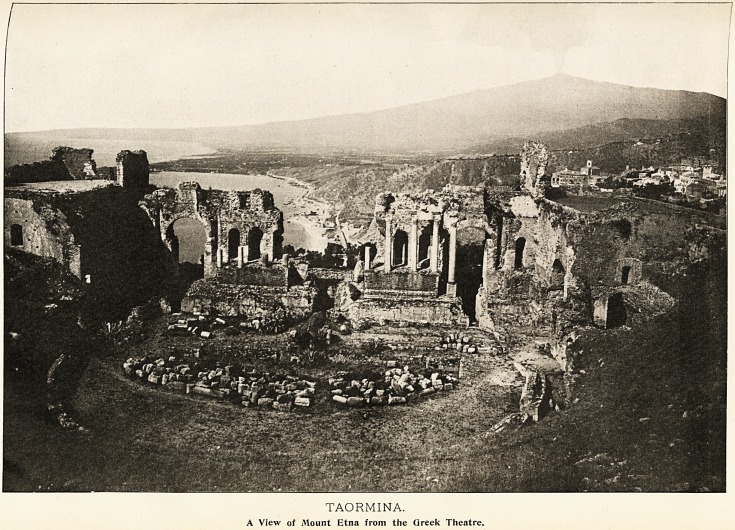# "The Hospital" Nursing Mirror

**Published:** 1898-10-08

**Authors:** 


### "The Hospital" Nursing Mirror.

Being the Nursing Section of "The Hospital."

[Contributions for this Section of "The Hospital" should be addressed to the Editor, The Hospital, 28 & 29, Southampton Street, Strand, London, W.C., and should have the word "Nursing" plainly written in left-hand top corner of the envelope.]

### News from the Nursing World.

### DEATH OF THE QUEEN OF DENMARK.

All England is grieving with the Princess of Wales in the sorrow which has fallen upon her in the death of her mother, and nurses will not be behindhand in offering their warm sympathy to one who is ever so kind a friend to them. The ties of affection which bind together the members of the Danish Royal Family have been proverbially strong, and throughout the last weeks of the Queen of Denmark's life her children have gathered round her in loving devotion, her three daughters — the Dowager Empress of Russia, the Princess of Wales, and the Duchess of Cumberland— being seldom absent from her side. All the sons and daughters, as well as the aged King, who had persistently refused to leave his wife through her last hours, were present when the Queen passed peacefully away at half-past five on Thursday morning.

### THE EMPRESS FREDERICK AT THE FRIEDRICHSHAIN HOSPITAL.

Just before starting for England the Empress Frederick paid a visit to the Friedrichshain Hospital at Berlin. She was accompanied by the Lady Superintendent of the Training Home of the "Victoria Sisters," and made quite a long stay in the women's surgical ward, speaking a few words to nearly every patient. On leaving the hospital a little girl presented Her Majesty with a bouquet of wild flowers, which she graciously accepted.

### LONDON SCHOOL OF MEDICINE FOR WOMEN.

It is announced that the Skinners' Company have given a further donation of 100 guineas in aid of the building fund of the London School of Medicine for Women. The first block of the new building, opened by the Princess of Wales in July, is now completely fitted and in use. The Helen Prideaux scholarship of £50 has been awarded to Mrs. Hamilton Williams, M.B., for an essay upon "Studies in Tuberculosis."

### ON POOR LAW NURSING IN NORTH WALES.

At the Poor Law Conference recently held at Menai Bridge an interesting paper on "Nursing in Workhouses and Among Outdoor Poor" was read by Miss A. B. Evans, late a guardian of St. Asaph Union, and a daughter of Archdeacon Evans, of St. Asaph. Miss Evans thought there was a slow improvement in North Wales in the matter of good nursing, but she regretted that in many instances guardians not only refused to support the claims of trained nurses, but even opposed grants being made to them. She spoke of the need for new infirmaries, built away from the workhouses, and touched on the difficulty of obtaining Welsh-speaking nurses. Guardians rightly insisted that the nurses in Welsh-speaking unions should be able to talk to their patients in their own tongue, but they in no way encouraged the associations formed to train such nurses for them. Miss Evans urged the necessity of offering trained nurses better wages and food, and of lessening their hours of work. She deprecated the unwillingness of guardians to subscribe, as they had a right to do, towards parish nurses for the outdoor poor, and suggested that the grants given at present by county councils to the St. John's Ambulance Society should go in support of trained nurses. In the discussion which followed Miss Evans' paper, the cause of the trained workhouse nurse was pleaded by several speakers.

### HYGIENE AT THE CHURCH AND SANITARY CONGRESSES.

At the Church Congress, held last week at Bradford, Miss Alice Ravenhill read an interesting paper at one of the sectional meetings on "The Keeping of the Home," dealing with matters hygienic and sanitary. She illustrated her address with a number of practical hints upon household management, expressing her conviction that the greatness of no nation can be secure that is not built upon a pure and wholesome home life. Miss Ravenhill had a busy week, for on Friday she was one of the speakers at the Sanitary Congress at Birmingham, and to her fell the honour of being the first woman to read a paper before the section of sanitary science and preventive medicine, lady speakers on previous occasions being only admitted to the domestic science section. Her subject was "The Position Assigned to the Teaching of Hygiene in our Elementary Schools." In this respect we are in England far behind other countries, only about 1 per cent. of the boys and girls in the elementary schools being given an opportunity of instruction in this important subject. Most sensible people will agree with Miss Ravenhill that "were ratepayers more alive to their own interests, educational and sanitary, it is conceivable they would demand, *en masse*, that a definite proportion of the huge sum they annually contribute for educational purposes should be used to inculcate a practical knowledge of health laws."

### WORKHOUSE NURSES.

The problem how to provide proper nursing for the sick in workhouses has much altered its point of view since the issue of the order of the Local Government Board by which the employment of paupers as nurses was put a stop to. The difficulty now is to get the nurses; for the nursing of paupers is not an altogether attractive occupation, and requires, if it is to be well done, a motive which has but little relation to the earning of a comfortable living. In the report of the Local Government Board, which has just been issued, Mr. Bagenal, one of the inspectors, complains of the difficulty of obtaining nurses for workhouses. "Good salaries," he says, "are offered, but there are no candidates." "What seems to be wanted," he adds, "is a new class of nurse, whose bond will not be merely the cash nexus, or the desire for the excitement of a profession, but who will partake more of the original type of Miss Nightingale, with the additional qualities of modern scientific training. If religion and science could join hands for the production of a new class of nurse the nursing difficulty might, perhaps, be solved. The evolution of this kind of nurse is probably reserved for the twentieth century." We are not quite sure about this. The Nightingale type of nurse works wonders. It is a reforming influence, it stirs the pulse of the nation, and it leads to the abolition of great abuses. It is hardly, however, the type of nurse which bumbledom is asking for; and, much as we should like to see an example of the type planted in every workhouse in the country, we do not think that the comfortable complacence of many boards of guardians, nor the happiness of the clerks at the Local Government Board, would be thereby promoted. If there was one thing which Miss Nightingale did better than another—except always the pouring-out of human sympathy in the presence of human suffering—it was the reform of abuses and the rectification of what was wrong, and we cannot shut our eyes to the fact that it is something wrong in the workhouse system that is at the root of the difficulty about the nurses. The workhouse master is king within the workhouse; the workhouse matron, by exigencies of the law, must be his wife; and, under these two officials, so curiously linked together in their interests, although in their duties separate and distinct, the nurse is placed. If the nurse were responsible only to the doctor and the committee, we doubt if there would be much difficulty in obtaining nurses.

### LEGAL NEWS.

THE following paragraph appeared in the *Standard* of the 29th ult.: "Miss Beattie, attired in a nurse's uniform, applied in person for assistance to reopen the action of 'Beattie v. Cullingworth,' which she had brought to recover damages against Dr. Cullingworth, of St. Thomas's Hospital, for operating upon her in a manner contrary to her instructions. She said she had written to the Home Office, and she denied that the Official Solicitor was instructed to appear in the case. She had no means, and had done nothing since the trial. Mr. Justice Channell explained to the applicant how to sue *in formâ pauperis*, which she might do if she had any further cause of action." We would remind our readers that for some time past Miss Beattie has been acting as secretary for a society calling itself the "Society for the Protection of Hospital Patients."

### BIRKENHEAD DISTRICT NURSING SOCIETY.

A PUBLIC meeting is about to be held in support of the Birkenhead District Nursing Society (affiliated with the Q.V.J.I.) at which it is announced that Princess Christian will be present. An augmented subscription list should be the immediate practical result of thus making more widely known the good work and the needs of the society. Four Queen's nurses are now at work amongst the sick poor of Birkenhead, and great is the need for their services.

### THE PASSMORE EDWARDS' CONVALESCENT HOME, LIMPSFIELD CHART.

IT is perhaps not so generally known as it might be that private patients are admitted to the beautiful convalescent home built for Charing Cross Hospital by Mr. Passmore Edwards on Limpsfield Chart, surely one of the finest sites in England. The position is ideal. The home stands on a plateau, well sheltered from cold winds, and facing a really glorious view over Kent, Surrey, and Sussex, the South Downs, Leith Hill, Hindhead, Crowborough Beacon, and many another well-known landmark being included in the wide landscape. The rooms provided for paying patients are charming, all facing the sunny south and the grand panorama outside. They are most comfortably and prettily furnished, with every regard to convenience. There is a private sitting-room and dining-room for the joint use of these invalid guests. The charges, including board, but not medical attendance, range from two guineas to three guineas for one person. Certain variations are made in the charges when two persons share the double rooms. Further information can be had from the secretary of the hospital, or from the matron at the home. A pleasanter place in which to "convalesce" it would be hard to find.

### LADIES V. GENTLEWOMEN.

THE correspondence on "Ladies v. Gentlewomen," which has been pursuing its course in the columns of the "Mirror," must have given matter for thought to many of our readers who are interested in the general development of nursing, and who are, perhaps, watching with feelings of something like regret the evolution of the modern nurse. It is, on the whole, hopeful to gather from the various letters that the view taken of the matter has been a broad one, and that the narrow and exclusive spirit which would make social position rather than general suitability a test of a woman's fitness for nursing has not many supporters. Indeed, in this democratic age it is difficult to see how it can be otherwise. There is something singularly undignified in this fighting for titles, and titles, too, which have come to retain little or nothing of their original significance. The "lower class" of one generation rises through education into the "higher class" of the next, and by a similarly natural process the fittest women attain to the higher posts in the nursing profession, the fittest women for such posts being necessarily the best educated and the most refined. Let us level up, not down; it is surely for the "ladies" who find their way into our hospitals for training to use their influence, not to exclude their less privileged sisters, but to aid in the evolutionary process by precept and example.

### SHORT ITEMS.

A SUCCESSFUL entertainment on behalf of the Radcliffe and District Sick Nursing Association was held the other day at the Queen's Hall, Radcliffe.—On Saturday, October 1st, a big demonstration in aid of the Nursing Association and the Accident Hospital took place at Barry, including a parade and a grand concert in the evening.—Mrs. Neufeld, accompanied by her daughter, has sailed for Naples on board the *Austral*, whence they proceed to join her husband. Mrs. Neufeld gave up her duties at the Northwich Isolation Hospital last week. She has been the recipient of hundreds of letters of congratulation.—The third annual meeting of the Maldon and Heybridge Cottage Nursing Association was held the other day, when many pleasant things were said of Nurse Blythe's good work amongst the sick poor. It is proposed to increase her salary.—The foundation-stone of a new Nurses' Institute was recently laid at Wombwell by Mrs. A. R. Garland, of Welham Hall. The institute has been built in commemoration of the Diamond Jubilee.

### Nursing in Diseases of the Throat, Nose, and Ear.

By MACLEOD YEARSLEY, F.R.C.S.Eng., Assistant Surgeon to the Royal Ear Hospital, Surgeon-in-Charge of the Department for Diseases of the Throat, Nose, and Ear, the Farringdon General Dispensary, Hon. Surgeon for Diseases of the Throat and Ear, the Governesses' Home, &c.

### IV.—OPERATIONS ON THE NOSE AND NASOPHARYNX.

THE preparation of patients for operations upon the nose and nasopharynx needs but few remarks. Their general preparation does not differ from that for other surgical operations, with the essential principles of which it is presumed the reader is well acquainted from her training as a general surgical nurse.

These operations may be divided into three groups: (1) Those upon the nasal cavities, (2) those on the accessory sinuses, and (3) those on the nasopharynx.

1. Operations upon the nasal cavities are done either upon the septum or the turbinate bones, or for the removal of new growths or foreign bodies. They are performed mostly through the natural passages, but it is at times necessary to considerably extend the operation in order to afford more room to the operator.

Many of the smaller operations are done in the consulting-room, and scarcely require the additional services of a nurse; among these minor procedures being the application of the galvano-cautery, the removal of spurs, small hypertrophies, &c. Little need, therefore, be said concerning them, but a word or two with regard to the preparation of the galvano-cautery or the threading of snares may not be amiss. A nurse who takes an intelligent interest in her work, who is quick and observant, and understands the little details of these small things is of much value to the specialist in his out-patient room. It saves him much time and trouble to have his cautery, snares, &c., prepared for him, and he is able to get through his work the quicker for such help. Most hospitals now possess the electric light, and are fitted with rheostats or transformers to utilise the current from the main for the cauteries. The nurse should ask to be shown the working of these apparatus; to enter into it here would be beside the province of these articles and would take up too much time and space. The handle used as a rule for galvano-cautery work is Schech's, and to it may be fitted various points or snares. When asked for the galvano-cautery, the nurse should inquire of the surgeon the kind of point he wishes fitted, and when it is arranged she should test it before handing it to him. The heat used should be sufficient to make the point a cherry-red colour, anything stronger is too intense, and causes pain and hæmorrhage. When finished with, the cautery is cleansed by simply turning on the current and burning away any tissue, &c., attached to the point. Snares should always be fitted ready with wire before the surgeon commences work, and, when they are used, fresh wire should be fitted with as little delay as is compatible with the purification of the instrument.

In removing spurs, various forms of instruments are used, the commonest being, perhaps, Bosworth's saw. For the removal of hypertrophies of the turbinal bones, nasal scissors, the snare, cutting forceps, or Carmalt Jones's "spokeshave" may be required. As the bleeding in these operations is nearly always very profuse, one of the nurse's most important duties is to see that the patient is properly prepared with towels, or mackintosh sheeting, to prevent the soiling of his clothes. Plenty of cotton wool should be at hand, as well as armed probes. When the surgeon has finished he will require to plug the nose, and for this strips of double cyanide gauze, soaked in 1 in 40 carbolic, should be ready for him, with forceps and probe to adjust them. These strips should be about twelve inches long by half to one inch broad, and, as such plugs are constantly employed both in nose and ear work, it is well that a large stock should be kept in 1 in 40 carbolic ready for use. Instead of plugging the nose with gauze, however, I now use, as do most of my colleagues at the Royal Ear Hospital, the splints of soft red rubber introduced by Mr. Lake.*

Passing now to operations under general anæsthesia, the patient should be prepared as for any general surgical operation. It must be borne in mind that in nearly all operations on the nasal cavities the hæmorrhage is free, and the nurse should therefore see that there is always plenty of cotton wool, swabs, hot water, &c., at hand. A square of mackintosh sheeting and three or four towels soaked in 1 in 20 carbolic, should also be ready. If the patient is a woman, it is well that her hair should be done up in a secure and compact coil, and well covered with a waterproof bathing cap or towel, adjusted turban-wise before the operation. Attention to this small precaution saves much trouble afterwards in cleansing blood-soaked hair. Other preparations for operation are much the same as in any surgical proceeding; there should be plenty of bowls, the instruments should be placed ready in a flat tray, needles, pressure forceps, snares, and cutting instruments being in separate dishes, and there should be a plentiful supply of whatever antiseptic the surgeon is in the habit of using. Some rhinologists like to have the nose douched before operation with an alkaline wash, to clear away any mucus, &c. The method of carrying out this measure has already been described in our second article. With regard to dressings, the surgeon should be asked what he wishes prepared, and everything should be ready to save the annoyance of delay. Points relating to the chief operations will now be shortly reviewed, and the instruments required for each detailed.

### A.—OPERATIONS ON THE NASAL CAVITIES.

1. ON THE TURBINAL BODIES.—These may be either complete or partial turbinectomies. The former (if done under a general anæsthetic) will require the following instruments: mouth gag, Carmalt Jones's "spoke-shave" nasal forceps, wool-holding probes. There should be ready plenty of armed probes and cotton wool. Peroxide of oxygen is used by some surgeons as a hæmostatic.† It is best kept in small bottles (half ounce) in a solution of twenty volumes, and one or two such bottles should be at hand, but not unstoppered, until required. The hæmorrhage in turbinectomy is considerable, and the remarks already made as to hair, &c., specially apply here. Plenty of the strips of gauze already described for plugging should be provided.

Partial turbinectomies are mostly done under cocain or eucain. The removal of posterior hypertrophies, however, is best performed under general anæsthesia. The instruments required are few, viz.: Snares, mouth gag, finger guard (an indiarubber finger stall with the tip cut off makes a very efficient guard against the patient's teeth), sponges on handles. The snares should be at least two in number; it is wiser to have three or four ready prepared in case the wire should break. As regards the mouth gag used, it may at once be said that the ordinary Mason's gag (with which the nurse must already be well acquainted) is sufficient for the purposes of all these operations. The sponges should be small and mounted firmly upon their holders; they are to be used for sponging out the nasopharynx. The best form

* *The Journal of Laryngology.* August, 1898, page 386.

† Gellé, Congrès de la Société Française d'Otolog.e, &c. May, 18[illegible]6

of holder is the two-pronged variety having the securing ring fitted with a bayonet catch.

2. Operations upon the Septum.—Operations upon the nasal septum comprise the removal of spurs and other outgrowths and the straightening of deviations. Spurs may be dismissed at once; they are practically always removed under local anæsthesia. The operations for remedying septal deviations are several in number. Forcible rectification is done with Adams's or Walsham's forceps, and involves fracture of the bony part of the septum. Specially-constructed plugs, or the rubber splints already referred to, are afterwards worn for some days. The instruments required are: Nasal speculum, probes, Walsham's forceps, nasal forceps, armed probes, nasal saw, nasal knife (these should be included in case the operator finds it necessary to remove any ridges), strips of gauze for plugging.

Asch's operation has been done both under a general and a local anæsthetic. Special instruments are required, namely: Separators (two, one sharp, one blunt), scissors (two pairs, straight and curved), compressing forceps (two pairs, with long and short beaks), hollow vulcanite splints. These instruments are of special form, the scissors cutting with one blade only, and space is too limited to describe them here. Armed probes will be wanted beside, and a nasal speculum and nasal forceps should also be put out. Hæmorrhage is best checked by means of cotton wool, iced aseptic water, or hydrogen peroxide.

There are various other operations for deviations of the septum, but those just given are the most typical and best known.

3. The Removal of Tumours.—The great majority of nasal tumours can be removed under local anæsthesia with cocain or eucain. Occasionally, however, the growth may be sufficiently large to require some additional operation in order to remove the whole growth properly, such as division of the soft palate, or resection of some of the bones of the face. Such operations as these are often performed by a general surgeon, and need not be dwelt upon here; probably the nurse has already seen them during her hospital training.

B.—Operations on the Accessory Nasal Cavities.

The most important of these procedures are the opening of the frontal and maxillary sinuses.

1. Maxillary Sinus (Antrum of Highmore).—This sinus is frequently opened and drained for purulent collections (empyema). Preliminary tapping through the nose for exploratory purposes may be done under cocain anæsthesia, the instrument used being a Krause's antrum trocar. This is a curved canula with a trocar and guide for entering the antrum through the outer wall of the nose just below the inferior turbinate body. In draining the sinus under general anæsthesia, it is opened either through a tooth socket or by piercing the upper jaw just above the canine tooth. The instruments required are: Antrum drill and syringe, curette, mouth gag, forceps, probes, cotton-wool swabs, antrum drainage tubes. A bowl of whatever antiseptic the operator uses for washing out the cavity should be ready. The antral drain-tubes used are of various patterns; a common form is made of silver wire wound spiral fashion into a tube.

2. Frontal Sinus.—This cavity is reached by an incision through the eyebrow, a drain introduced through its exit into the nose, and the wound sutured. The instruments required are: Scalpel, small trephine (some operators use a drill or a chisel and mallet), curette, long probe, drainage tube, forceps, needles, sutures, cotton-wool swabs.

C.—Operations in the Nasopharynx.

The most frequent operation done upon the nasopharynx is for adenoids. So much has been written upon the subject that one might almost say the methods of operation are legion. Some use the finger nail or an artificial scraper only, others use forceps, or various shaped curettes. Some prefer no anæsthetic, others gas, many chloroform, some ether. Operators also differ much as to the position of the patient. Some prefer them lying down with or without the head depending over the edge of the couch; others like to operate with the patient sitting up. But whatever the position or plan of operation affected by the surgeon the principles are the same. The nurse should bear in mind that the hæmorrhage is free and the directions as to covering the hair, &c., already described should be attended to. In preparing for the operation in a private house it is a good plan to spread the floor with old newspapers to prevent soiling the carpet. Plenty of hot and cold water, bowls, and basins should be provided, and the following instruments should be put out: The particular instrument used by the operator, Lowenberg's forceps, Gottstein's curettes, or some other pattern of curette, finger protector, sponges on handles, mouth gag. It is also wise (and this remark applies to all operations on the nasopharynx) to have the instruments required for tracheotomy (to be detailed in a future article) at hand. The method employed by my colleagues and myself at the Royal Ear Hospital is as follows: The patient lies in the dorsal position with the head partly over the end of the table. Gas is sometimes used for adults, chloroform for children. The mouth gag is adjusted, and the chief mass of growth is removed at one sweep with a curette provided with a cradle. Gottstein's curette is then used to scrape laterally, and the operation is finished with the nail, the finger being protected by an indiarubber sheath. As the Gottstein's curette is discarded for the nail the patient is turned over on to his face in order that the blood may have free exit from the mouth. I am convinced that this method of working is the best and most effectual.

The remarks already made regarding nasal tumours applies equally to those growing in the nasopharynx.

### Everybody's Opinion.

[Correspondence on all subjects is invited, but we cannot in any way be responsible for the opinions expressed by our correspondents. No communication can be entertained if the name and address of the correspondent is not given, as a guarantee of good faith but not necessarily for publication, or unless one side of the paper only is written on.]

The President of the Newark Hospital writes: My attension having been drawn, as President of the Newark Hospital, to a paragraph in your issue of Saturday last, casting certain reflections on our hospital, I feel I cannot allow your remarks to pass without a reply. The report of a child suffering from the effects of poison having been brought to our hospital and not being attended to is greatly exaggerated. The real facts are as follows: A child was brought to the hospital during the absence of our resident medical officer, on his rounds in the town visiting the out-patients, having had some poison given to it by mistake. It was seen by several of the nurses, and it did not appear to be much the worse, and the matron, not knowing the nature of the poison, did therefore, in the opinion of the Board of Management, the best thing she could under the circumstances, viz., sent it to the nearest doctor, there being three resident within a few hundred yards of the hospital. It was attended to by the first called upon, and recovered. The child was *not* in convulsions, nor had the mother to carry it all over the town in this state. I think before publishing a report such as you have done it would have been better to have ascertained the real facts of the case, and I must ask you to send me the name of your correspondent, and also, in fairness to our staff, publish this letter in your next issue.

*** The point we urged was one of which the unfortunate occurrence referred to appeared to be a fair illustration, viz., that where institutions do not provide for the continuous presence of a house surgeon some arrangements should be entered into with neighbouring practitioners for help in emergencies.—Ed. *T. H.*

### Antiseptics for Nurses.

By a Medical Woman.

### XIX.—SOAPS (*continued*).—GLYCERINE AND ITS COMPOUNDS.

In addition to the ordinary soaps, there are various so-called "oleates" in use, these consisting of a combination of various metals with oleic acid, this latter being a fluid acid which is usually not quite pure, and which is obtained by either the saponification of olein (a fluid oil obtained from olive oil), or by treating fats with superheated steam and subsequently separating the solid fats by pressure. Oleic acid is a pale yellow liquid, almost odourless and tasteless, and of only slightly acid reaction, but when exposed to the air it becomes brown-coloured and also acid. It is almost insoluble in water. The oleates mix well with other fats, are very readily absorbed by the skin, and are said to be non-irritating, and, consequently, are very useful for conveying various drugs. The two best known are oleate of mercury and oleate of zinc, but other metals have been incorporated. It has been proposed, on account of the above properties, to incorporate oleates with soaps, especially as a means of conveying the disinfectant; but as the efficiency of soap depends on its solubility, and oleates are insoluble in water and merely form a curdy precipitate which is probably inert, and as also the metallic oleates are insoluble in water, it is evident that the addition of oleates to disinfectant soaps will not increase their efficacy. It has also been shown by experiments that metals dissolved in soda or potash would probably give better results than would the oleates. Mercury being such an excellent antiseptic, it was suggested that it would make a good disinfectant soap if it were possible to produce it without allowing it to form an insoluble oleate, which would have very little power as a germicide, and which also will not make a good lather; another drawback being the fact that any surface on which a mercurial preparation is used is liable to become blackened, and that organic matter is apt to reduce and make the mercury inert. One form of mercurial soap contains a combination of perchloride of mercury, ammonia, mercuric chloride, beta-naphthol, eucalyptus, and methyl salicylates, which are all incorporated in a dry state with a neutral soap, and are therefore supposed to be present in an unchanged state, and are said to be active at the time of using the soap in washing, but an oleate is formed more slowly and later.

None of the disinfectants belonging to the group either of "acids" of what are known as "halogens," viz., chlorine, bromine and iodine, can be used in conjunction with fats, and consequently they are excluded from antiseptic soaps, since the acids are neutralised by the alkalies of the fat, and the latter combine at once with the fat, and hence are useless. It is possible that some of the iodine and bromine compounds that have been introduced in large numbers of late years might be available for the purpose, but their irritating properties and disagreeable odours are a great drawback to their use.

The soaps that are most used medicinally are curd soap (made with soda and a purified animal fat consisting chiefly of stearin, and, when pure, being of a light grey colour and nearly inodorous), used in various plasters; hard soap, known also as sodium oleate, and made with olive oil and soda, and used in liniments; and soft soap, or potassium oleate, made with olive oil and potash.

Arsenical soaps have been proved to contain only an infinitesimal amount of arsenic, and are consequently useless for antiseptic and disinfectant purposes. If used sufficiently frequently, however, even this small amount of arsenic might have some effect on the skin.

Amongst the chief varieties of medicated soaps which are manufactured may be mentioned those containing carbolic acid, petroleum, mercurial salts, sulphur, borax, camphor, iodine, and chlorine; but thanks to the researches of Dr. Rideal, to whom we are greatly indebted for much information, it is now possible to distinguish between the merits of these various soaps.

Glycerine in itself cannot be regarded as an antiseptic in the true sense of the word in that it does not exert any bactericidal action on even the weakest micro-organisms, but when pure and kept carefully and all air excluded it is an aseptic substance, being free itself from all micro-organisms, and hence is an excellent application when it is required to merely exclude the air and act as a demulcent. Glycerine is formed during the manufacture of soap, and is a sugar-like substance which can be obtained from most natural fatty bodies when acted on by alkalies, such as potash or soda, or similar reagents, which decompose the fats; as a result, water is taken up, and glycerine and an alkaline salt of some particular acid, according to the nature of the special fat used, are formed. This process is commonly called saponification, because this same decomposition occurs in soap making, as soap is a mixture of these various alkaline salts in various proportions according to the quality of the soap and the purposes for which it is required. Thus in the ordinary process of soap making, the complementary product, glycerine, remains dissolved in the aqueous liquors from which the soap has been separated, and is either thrown away, or is itself separated and carefully purified. Glycerine is a colourless substance, very soluble in water; it does not solidify except at a very low temperature, and is readily absorbed by the skin when unbroken, and consequently is very useful as a medium for inunction and as an addition to lotions. It can be combined with antiseptic powders, such as iodoform, boracic acid, creolin, &c., to form emulsions, and with carbolic acid, alum, &c. It is said to possess sufficient antiseptic power to delay putrefactive changes in meat and albumin that have been immersed in it for long periods of time. Glycoformal is the latest preparation of glycerine in combination with formaldehyde, and is another method of preventing that polymerisation of formaldehyde to which we have already referred. To bring about the combination an apparatus is constructed consisting of a vessel in which the water is boiled, and as the steam rises it is collected in a reservoir containing 40 per cent. of formaldeheyde and 10 per cent. of glycerine; these combine and form glycoformal, which is used for disinfecting rooms by merely arranging for four pipes to pass from the reservoir out into the room. Glycoformal is heavier than the air, and consequently sinks, and should therefore enter high up in the room, which is quickly filled with the glycoformal, and it is said that all microbes are destroyed in three hours. Schlossmann claims for it the following advantages, viz., absolute sterilisation, the closure of all openings and cracks is not necessary, there is no danger of explosion, the method is cheap, and the total disinfectant powers of the gas are obtained.

Glycerine has been used for sterilising instruments, and for this purpose has been heated to 140 deg. C., but it has not been possible to continue its use, as it decomposes when subjected to great heat, and causes an intolerable smell.

Glycerinum saponatum is made by mixing together soda soap and neutral cocoanut oil, in the proportions of 8 to 20 per cent. with 80 to 90 per cent. of glycerine, forming a yellowish-white, quite inodorous, more or less elastic mass, which melts at the temperature of the body, and is readily soluble in either cold or warm water. It dissolves with great readiness a large number of substances, and holds others which are insoluble, such as powders, in suspense, and hence is an excellent medium for the inunction of drugs, and this constitutes its chief advantage.

### Training in the Provinces.

(Continued from page 240, Vol. XXIV.)

### ROYAL HALIFAX INFIRMARY (100 Beds).

### Terms of Training.

Probationers are received for training at the Royal Halifax Infirmary between the ages of 22 and 30 years. If satisfaction is given during the first trial period of one month, the candidate is allowed a further probation of two months, at the end of which time an agreement is entered into for three years' training, the three years dating from the end of the first month. Salary begins the first year at £12, increasing to £14 during the second, and to £16 during the third year. Probationers breaking their engagement are required to pay a forfeit of £6 and to give three months' notice.

Staff nurses' salaries range from £20 to £23; sisters from £25 to £30. Indoor uniform is provided for all the staff by the hospital, nurses being expected on leaving its service to refund the present value of their dresses, &c.

Night duty is taken by nurses in their second and third years in alternate periods of three months.

Only nurses who have completed three years' training and gained satisfactory certificates are eligible for appointment on the permanent staff of the infirmary.

### Hours On and Off Duty.

The length of the average working day is estimated at 10½ hours for probationers, and at 10 hours for charge nurses or sisters. Two hours off duty time are allowed daily, probationers having a half-day on Sundays, and staff and charge nurses being, in addition, given leave from 5 to 9 p.m. one day in the week. A fortnight's yearly holiday is allowed after the first year of service.

### Meals.

All meals for the day nurses are served in the nurses' dining-room, with the exception of the early luncheon, which is taken in the ward kitchens. The hours for meals are as follows: Breakfast, 6.30 a.m.; lunch, 9.30 a.m.; dinner, 1 to 2 p.m.; tea, 4 to 5 p.m.; and supper, 9 p.m. No "allowances" are given into the nurses' hands.

### HEREFORD GENERAL INFIRMARY (107 Beds).

### Terms of Training.

The course of training offered at the Hereford General Infirmary is two years, a certificate being granted at the end of that time. Candidates should be between 20 and 28 years of age. No premium is asked, salaries beginning at £8 for the first year, and increasing to £10 during the second year. Charge nurses' salaries range from £25 to £27 per annum. Indoor and outdoor uniform is provided by the hospital for all the staff, and laundry also.

Probationers are eligible for appointment as charge nurses on completion of two years' training if they show themselves to be in every respect suitable for promotion.

### Hours On and Off Duty.

Probationers are on duty from 7 a.m. to 2 p.m., and from 4.30 to 9 p.m., this giving 2½ hours off duty daily. Charge nurses are on duty on alternate days from 7.30 a.m. to 9 p.m. and from 7.30 a.m. to 4.30 p.m. Time for meals must be deducted from these working hours. One whole day off duty is allowed each month to nurses and probationers. Night nurses may, if they like, take four days every three months instead of one every month.

Night charge nurses are on duty from 9 p.m. to 7.30 a.m., night probationers from 9 p.m. to 8.30 a.m.

Three weeks' holiday is allowed per annum.

### Meals.

All meals except the early lunch are served in the nurses' dining-room. Breakfast is from 7 to 7.30 a.m., lunch is taken at the convenience of the nurses, dinner is served from 12.30 to 1 p.m., tea from 4 to 5 p.m., and supper from 8.30 to 9.30 p.m. Allowances of butter, sugar, &c., are given out only to the night nurses for their midnight meal.

### HUDDERSFIELD INFIRMARY (100 Beds).

### Terms of Training.

A three years' course of training is offered to probationers at the Huddersfield Infirmary, candidates being required to undergo a trial of three months before they are finally accepted. No premium is asked, salary commencing the first year at £10, rising to £15 the second year, and to £20 the third year. Charge nurses' salaries begin at £25 per annum, increasing to £30. No probationers trained at the infirmary are eligible for appointment upon the permanent staff until they have satisfactorily completed the full term of three years' training, at the conclusion of which only are certificates granted. Lectures are given to the nurses during the autumn and winter months by the house-surgeons. Indoor uniform and laundry are provided by the hospital for all the nursing staff. The second and third year probationers take night duty and day duty alternately for six weeks at a time.

### Hours On and Off Duty.

Nurses' hours on duty are from 7.30 to 9 p.m., with two hours for recreation daily, and time for meals, and a half-day once a week. Alterations and rebuilding of the infirmary are at present in progress; there will soon be a new nurses' home, and the rules will also be revised before the close of another year. Night nurses are on duty from 8.45 p.m. to 8.30 a.m.

### Meals.

All meals are served in the dining-room, food allowances being only made to the night nurses for their midnight meal. The hours of meals for day nurses are as follows: Breakfast, 7 and 7.45 a.m.; dinner, 12 and 1 p.m.; tea, 4 and 4.30 p.m.; supper, 9 p.m.

### Appointments.

Sir Titus Salt's Hospital, Saltaire.—Miss Hannah Mitchell has been appointed Matron of this hospital. She received a three years' training at the Bradford Royal Infirmary, and has since acted as sister of the men's accident, and of the children's surgical ward at the same institution. She gained the bronze medal on completing her training, and later the gold medal of her training school.

Caterham Cottage Hospital.—Miss M. L. Smith has been appointed Matron of this institution. Miss Smith was trained and took her certificate at the Evelina and King's College Hospital, and was for two years out-patient sister at the Victoria Hospital for Children, Chelsea. Since December, 1896, she has been assistant matron at the Royal Berks Hospital, Reading.

County and City of Cork Lying-in Hospital.—Mrs. Blunden has been appointed Matron of this hospital. She was trained at the North Infirmary and City of Cork Hospital, and at the Rotunda Hospital, Dublin (Maternity Department), at which latter hospital she was for some time as staff nurse to the Gynæcological Department.

Woodilee Asylum, Lenzie, N.B.—Miss E. J. Rae has been appointed Matron to this institution. Miss Rae was trained at the Westminster Hospital, and has held appointments as home sister at the Brompton Hospital, and as matron of the District Nursing Home, Liverpool.

### Nursing in Paris Hospitals.

### C.—THE NURSING SISTERS.

### IV.—COMPARATIVE COST.

I FEAR that, after all, the final judgment of the Paris public on the question as to whether the hospital laicisation has been beneficial or injurious will be given not from the side of the patient, that is as to the respective capacity of Sisters and laics as nurses, but from the point of view of the taxpayer, as to whether the laicisation has been economical or expensive.

Judged from the budgetal standpoint, there seems to be no doubt that laicisation has been little short of a public disaster for the French capital. I have referred at length in my Series A to the constantly increasing demands of the new laic employees in the matter of pay and allowances. In the hospital budget of the Municipal Council for 1898 we have again a great increase proposed, that of 200,000 fr. Let us see how the ediles of the Hôtel de Ville propose to get the money.

The hospital budget is already in such a parlous state that the Council hardly cared to add the 200,000 fr. to the aggregate expenses. Savings had to be made, and at the expense of the patients as follows: Wine cellar, 50,000 fr.; provisions, 32,000 fr.; heating and lighting, 10,000 fr.; washing 5,000 fr.; bedding, linen, and clothing, 80,000 fr.; instruments, 5,000 fr.; medicines, 5,000 fr. This nearly makes up the increase in wages, but was almost needed if economies had to be made for the 182,000 francs required for the increased price of wheat before Mr. Joseph Leiter's great boom had burst.

Of course, the Municipal Council was not responsible for the injury which, in common with so many millions of sufferers throughout the world, the hospital patients of Paris had to suffer this year on account of the threatened Leiter tax, but it is decidedly an unpropitious moment for increasing the nurses' pay. The budget of the Assistance Publique is verging towards bankruptcy, for when the Assistance Publique sells a house or land, they are obliged to invest the proceeds in the state rentes. Nine-tenths of the dividends of such investments they may use as income; the other tenth has to be capitalised for an emergency fund. During the last ten years the Assistance Publique has been obliged to sell eleven million francs of this fund to meet the annual deficiencies in their budget. In a year or two this fund will have entirely disappeared, and they will have to fall back on the sale of legacies, and finally will be bankrupt. There is a definite method in the apparent madness of the Municipal Council in this respect. The Municipal Council is deliberately pushing the Council of the Assistance Publique to the wall, desiring the eventual bankruptcy of the latter body. When all the funds and legacies are exhausted, and the only income available is the municipal taxes, the Municipal Council will get complete control of the hospital administration. As a fact, the control is increasing year by year. In 1896 ten members of the Municipal Council were added as *ex officio* members to the Conseil Superieur of the Assistance Publique, making one-third of the whole. Though the Municipal Council is only supposed to give "advice" on the budget of the Assistance Publique, this "advice" amounts to "orders." The Government have supreme control of the budget, but their motto is "anything for a quiet life," and does not care to be always fighting the pugnacious Parisian politicians.

At present, apparently, the partisans of laicisation have flung to the winds all idea of either concealing or minimising the increased expenditure from the change, basing their case entirely on the allegation of more efficient service. As late as 1892, however, the majority of the Municipal Council took great pains to put forward elaborate financial pleas for the contention that the actual cost had, after all, been comparatively little. Some extraordinary claims were advanced to support this contention. Thus, M. Navarre, the reporter of the budget, claims the suppression of chaplains' salaries as a part of the economy of the expulsion of the nursing Sisters, a rather far-fetched plea. In a keenly-written little pamphlet, M. Henry Alpy, one of the minority in the Council, pulled to pieces the contentious figures advanced in the budgets of 1890, 1891, and 1892 by MM. Strauss, Navarre, and Risler, the respective reporters. As M. Alpy's figures have since, I believe, never been seriously impugned, and as he announced his purpose in issuing his pamphlet to be to prevent the stifling of his motion for an inquiry on the matter, I cannot do better than to summarise the schedule of M. Risler, the reporter, and the counter-schedule of M. Alpy.

Two or three laicised asylums are included in the statement of both parties. To avoid complicating matters I retain the statements intact. The asylum figures do not affect the comparative results to any appreciable degree.

Firstly, M. Risler announces the "augmentation of expenses from laicisation" as follows:—

| Establishments. | Date of laicisation. | No. of Sisters. | Allowances *vestiaire* (francs). | No. of lay matrons, &c. | Wages (francs). | Increased expense (francs). |
|---|---|---|---|---|---|---|
| Pitié ... | ... Oct. 10, '89 | 24 | 4,800 | 30 | 12,420 | 7,620 |
| Charité ... | ... Jan. 23, '88 | 18 | 3,600 | 26 | 39,900 | 23,800 |
| Saint Antoine | ... Aug. 1, '81 | 22 | 4,400 | 30 | 12,300 | 7.900 |
| Necker ... | ... Oct. 28, '86 | 19 | 3,800 | 21 | 10,800 | 7,000 |
| Cochin ... | ... Dec. 21, '85 | 26 | 4,800 | 24 | 11,900 | 7,100 |
| Beaujon ... | ... Oct. 1, '87 | 20 | 4,000 | 25 | 37,600 | 18,600 |
| Lariboisière | ... Sep. 15, '87 | 27 | 5,400 | 34 | 50,100 | 24,450 |
| Tenon ... | ... June 1, '82 | 21 | 4,200 | 36 | 18,100 | 13,900 |
| Laënnec ... | ... Dec. 1, '82 | 17 | 3,400 | 46 | 14,000 | 10,600 |
| Lourcine ... | ... June 1, '82 | 15 | 3,000 | 14 | 14,300 | 11,300 |
| Children's | ... Oct. 28, '86 | 26 | 5,200 | 31 | 16,000 | 10,800 |
| Forges ... | ... Oct. 28, '86 | 10 | 2,000 | 10 | 4,800 | 2,800 |
| Trousseau | ... May 1, '87 | 20 | 4,000 | 26 | 34,800 | 15,800 |
| Foundling | ... April 1, '86 | 27 | 5,400 | 34 | 23,500 | 18,100 |
| Incurables | ... Feb. 1, '85 | 62 | 12,400 | 40 | 21,000 | 8,600 |
| Menages ... | ... Jan. 1, '81 | 26 | 5,200 | 19 | 8,100 | 2,900 |
| La Rochefoucauld | Jan. 1, '81 | 11 | 2,200 | 6 | 2,640 | 440 |
| Totals ... | | 391 | 77,800 | 452 | 332,260 | 191,710 |

There are a few apparent discrepancies to explain in the above. Thus, I presume, at the Cochin Hospital there were two Sisters not "on the strength," as our soldiers say, otherwise the 389 Sisters (deducting these two extra ones) at 200 fr. each makes the total of 77,800 fr. of the ancient wage allowance. Then M. Risler allows for the cost of the Sisters' keep at four hospitals, where the new matrons were not fed, 62,750 fr.

viz., at the Charité, 12,500 fr.; at Beaujon, 15,000 fr.; at Lariboisière, 20,250 fr.; and at Trousseau, 15,000 fr., all of which, added to the 77,800 fr., makes 140,550 fr. for the Sisters, which, subtracted from the 332,260 fr. for the new nurses, gives the extra 191,710 fr. in the last column. Then, to this added cost of laicisation, M. Risler added the temporary items of 66,319·03 fr. for pensions to discharged Sisters (reduced to 57,690·50 fr. already in 1888), and the large figure of 522,773·65 fr. for building alterations required for the new nurses, of which 18,594·81 fr. was for entirely new creations, and the balance of over half a million francs for mere alterations of the Sisters' domiciles.

This admission of considerable increased cost by the mouthpiece of the laic party does not at all satisfy M. Alpy. He criticises the items in detail, and also the general plan as misleading. His own claims are summarised in two tables, the chief points of which I condense, as follows:—

| Establishments. | No. of beds before change. | In 1892. | Staff before change. | In 1892. | Expenditure (francs). Before change. | Expenditure (francs). In 1892. | Expenditure (francs). Increase. |
|---|---|---|---|---|---|---|---|
| Pitié ... ... | 709 | 714 | 127 | 138 | 35,960 | 52,300 | 16,340 |
| Charité ... | 516 | 584 | 109 | 131 | 33,200 | 75,700 | 42,500 |
| St. Antoine | 647 | 816 | 127 | 178 | 36,130 | 63,600 | 27,470 |
| Necker ... | 442 | 474 | 79 | 116 | 23,000 | 43,500 | 20,500 |
| Cochin ... | 378 | 506 | 92 | 117 | 25,900 | 42,600 | 16,700 |
| Beaujon ... | 432 | 480 | 102 | 130 | 30,900 | 76,400 | 45,500 |
| Lariboisière | 704 | 864 | 142 | 187 | 42,600 | 105,400 | 62,800 |
| Tenon... ... | 635 | 928 | 135 | 200 | 43,350 | 71,800 | 28,450 |
| Laënnec ... | 520 | 628 | 95 | 139 | 18,380 | 52,000 | 33,620 |
| Lourcine ... | 243 | 312 | 39 | 41 | 10,850 | 27,300 | 16,450 |
| Children's ... | 593 | 629 | 133 | 160 | 45,000 | 72,300 | 27,300 |
| Forges ... | 224 | 224 | 30 | 31 | 8,300 | 11,600 | 3,300 |
| Trousseau ... | 463 | 536 | 96 | 128 | 28,000 | 74,400 | 46,400 |
| Foundling... | 685 | 604 | 134 | 200 | 45,100 | 89,600 | 44,500 |
| Incurables... | 2,147 | 2,209 | 175 | 182 | 49,800 | 68,800 | 19,000 |
| Menages ... | 1,387 | 1,461 | 59 | 74 | 17,310 | 28,200 | 10,890 |
| La Rochefoucauld... | 246 | 245 | 28 | 24 | 6,820 | 9,000 | 2,180 |
| Totals ... | 10,971 | 12,214 | 1,702 | 2,176 | 500,600 | 964 500 | 463,900 |

In plain words, an increased accommodation of little over one-tenth has accompanied an increased expense of something approaching double. Considering the constant series of augmentations since 1892, noted by me in Series A, I think that we are well within the mark in putting down the direct increase in expense of laicisation, allowing for all increased accommodation, as fully 100 per cent. When the Parisian taxpayer fully realises this item of comparative cost, I believe he will be inclined to answer M. Alpy in the words of our old friend Mr. Puff, "Ha! thou hast touch'd me nearly!"

EDMUND R. SPEARMAN.

### "Nursing Notes" on Massage.

THE Editors of *Nursing Notes* have devoted a large amount of space in their October number to the subject of massage. There are a number of papers written by practising masseuses, treating the subject from various points of view, which contain much valuable advice and information. The objects and work of the society of trained masseuses are also dealt with. The success which has attended the formation of the society must be very gratifying to its founders; it has triumphantly achieved its aim of supplying the public with efficient and well-trained masseuses of the highest character, whilst at the same time effectively safeguarding its workers.

### Mediæval Practice in the Care of the Sick.

### IX.—FURNITURE AND PROCEDURE.

MANY medical and other books give directions about the position in which persons asleep should be allowed to lie, and particulars as to the best kinds of clothing, beds, ventilation, and general sick-room hygiene known to the authors. Scarlet night-caps are recommended, and also the practice of putting a "flock or clean woole" mattress over a feather bed that a patient "lie not too hot nor to colde, but in a temperance." Beds had much improved when, in the fourteenth century, a picture of a king and queen wearing their crowns during their night's rest, was drawn, and in the fifteenth and sixteenth centuries tester bedsteads and all the fittings were very like those of to-day.

Feather beds were much used during the epidemics of the sweating sickness, not always wisely one may infer, as a German doctor, writing in 1529, forbids their use in cases of this kind, though he insists strongly upon the necessity of preserving the patients from chills, recommending the practice of sewing the sick man's bed-clothes down to the edge of his bed. English physicians seem to have been regarded as authorities upon this plague, but an extract from "A Boke of Counsell against the disease commonly called the Sweate or Sweatyng Sickness," published in 1552, will serve to show that John Caius considered the lives of the sufferer were very much in the hands of their nurses. "In what time soever it taketh us," we must keep ourselves quiet in bed and "Oure kepers, friends and louers must also endeavoure themselvss to be handsome and dilygente aboute us, to serve us redilye at all tymes, and never to leave us during foure and twenty hours, but to look welle to us, that neither we caste of our clothes nor thrust out hand or foot duryng the space of the saide foure and twenty hours. For albeit the great danger be passed after twelve hours, or fourteen, the last of trial, yet many die after by too much boldness. . . . Whereby it is proved that without dout the hansome diligence or careless negligence is the sauving or casting away of many."

Nurses attendant upon these cases were required to administer drinks at stated hours; to pay careful attention to the symptoms and act accordingly; if possible, to keep the sick man from faintness by conversation; but if the patient did faint, in spite of all precaution, south windows might be opened with due carefulness, handkerchiefs steeped in vinegar and rose water applied to his nose, and the nurse was to place him on his right side, beat him with rosemary branches and shout to him until he recovered.

After cauterisation, says Lanfrank's "Science of Cirurgie" (recently edited by Dr. R. V. Fleisch Hacker from two MSS., one about 1308 and the other 1420), there may appear an ulcer, in which case butter, or cold grease, with cabbage leaves, which is still better, shall be laid thereon. Plasters of barley meal, roses, camphor, and vinegar were used in cases of dropsy; garlic, oil of lilies, camomile, and vinegar were applied to gathered ears; and linseed meal was as commonly used for poultices as it is to-day.

We read of linen bandages, in cases where constant renewal must have necessitated the application of them being entrusted by medical men to the patients' nurses. Indeed, deft womanly fingers were, no doubt, useful then as now in the manufacture of padded splints, belts for hernia, and other kindred contrivances.

### Minor Appointments.

ESSEX AND COLCHESTER HOSPITAL.—Miss Sophie E. Hooke has been appointed Night Sister at this hospital. Miss Hooke was trained and certificated at University College Hospital.

### Pictures from Paris.

By an English Nurse.

### An Operation.

The heat is excessive, the air still and tepid, and the long flower borders in the garden of the hospital blaze in the sunshine. Under the trees in the courtyards between the pavilions the children are lying or sitting, looking deliciously cool with their cropped heads and blue blouses.

The surgeon in his white holland overalls stands chatting at the ward entrance, the inevitable cigarette between his lips. Big, sloppy-looking nurses in list slippers go lounging about the wards, following the sharp directions given by the brisk *surveillante*, who sits busy at her table.

A man in black comes quickly up the centre road of this village of wards, and the place seems to awake to meet him.

"The Chief!" says the *surveillante* to the least sloppy of the nurses. "Is the operation room ready?"

Students in overalls group round the Chief as he makes his way to the tiny operating room. They are all gesticulating and talking. The anæsthetist takes his seat at the head of the table; the nurse puts the instrument trays ready; the chief and the house surgeon are scrubbing their hands.

"There, Jean; mount the table like a man!"

The slim little lad of thirteen gets on to the operating table and smiles somewhat nervously round at his nurse, who is leaving him here with strangers. But he is *brav garçon*, and yields himself up to the chloroform without a word or struggle.

How hot it is here in this little operating-room at the end of the ward! There is no space to move, though there are only seven or eight people present, and the smell of the chloroform mixes with the heat from the sterilising apparatus in the corner, and makes the air positively stifling. The operation goes on and on—one cyst is removed from the liver and then another, and then the surgeon makes a transverse section, and goes off exploring in the region of the spleen. Over fifty pairs of artery forceps are in use already, and as the surgeon moves them this way and that he looks as though he were making bobbin lace.

It is exactly two hours from the time of the first incision that the last stitch is put in and the dressing begun. Two students have gone off for the stretcher; the nurse is picking up the empty chloroform bottles; the chief goes off to report to the "director" that it has been a very grave operation. The boy recovers.

### A Meal.

The big clock clangs out noon, and "Mdlle. Anglaise," who has now learnt her way about the big, spreading, old hospital, goes off across courtyards and through archways to the refectory. The first breakfast division have just left, and the tables are being prepared for the second division. Beside a great hot tin stands a handsome Frenchwoman, a huge spoon in her hand. Each nurse picks up a plate and holds it out, and receives her piece of veal cutlet. There is another tin with potato purée, but vegetables here form a second course, and are not served with the meat. The third course consists of cheese. The refectory is large and bare, the floor is sanded, the only ornamentation on the walls is a notice of the forthcoming lectures at La Pitié. The nurses have all put on their white linen fichus to come to breakfast, but their ill-fitting blue gowns, and caps consisting of muslin Alsatian bows, are not smart-looking. At one table sit the hospital porters, the nurses sit at the next table, a third small table is reserved for the *premieres infirmieres*, or staff nurses as we should call them. The *surveillantes*, who answer to our sisters, live in a huge building in a corner of the grounds with their families—many of them are married women—and take their meals at home.

Before each nurse is a small decanter of wine. The long roll of French bread is passed round, and each cuts off a hunk. The food is very good and excellently cooked; it is not served with nicety nor eaten with nicety; but everything is clean. Through the great door at the end of the refectory you can see the big kitchen with its copper pans all shining and spotless. So far as food goes the Paris nurse has no cause of complaint.

### The Labour Room.

The maternity department is bran new, and has only been open a little over a year. It is a square pavilion in red brick and red tiles, there is a glass verandah all round, and a broad-leaved palm flourishes in the centre of the courtyard. Sitting under the verandah and looking over the red roof to the white houses and purple distances beyond, feeling the still heat which falls on the little garden, one thinks of Algiers and of all things Eastern and far away. One might be miles from Paris, so quaint is the effect. Off the verandah is a great big white tiled labour-room, with four beds in a row; two beds are occupied, and the patients lie naked from the breasts down while the work of the hospital goes on around them The midwives sit and chat and sew at the long table; the nurse wheels in the dressing stands from the wards, and proceeds to fill the glass bowls and jars. The students come in and sit on the table and chat also—one is in his military uniform. The porters pass in and out, and even the men from the central who are taking the inventory come and go with only a passing glance and a shrug of their shoulders at the women in labour. Such a thing as a screen is unknown in a Paris hospital. Presently the Chief comes in—a little gentle, kindly man, who says to each patient, "N'ayez pas peur, ma petite fille," and a breath of human feeling seems to come with him. But he goes again, and no one heeds the patients any more.

The heat is terrible; the midwives have removed their needlework and chatter to the courtyard, where the verandah shelters them from the afternoon sun. They are drinking beer and consulting a book of fortunes. It is four p.m.

"May I have some water? I have been here since nine o'clock and had nothing—nothing at all!" says the poor primipara on the second bed. Mdlle. Anglaise goes to the midwife in charge of the labour room and repeats this information.

"She must ask the nurse," says the midwife, tossing her pretty head on which the coils of hair are gathered high.

"There is no nurse there."

"Louise!" calls the midwife, "give the patient some tea."

"There is no tea—no tisane," replies Louise, "they are taking the inventory, and I cannot get anything."

"Oh, this inventory!" says the midwife; "that is why we haven't any chairs even to sit on."

"But cannot the patient have some water, then?"

"Louise, give the patient some water," calls the midwife shrilly. Louise comes lounging out of the examination room and empties someurine glasses down the doctor's basin; then she gives the patient a drink of water. Mdlle. Anglaise stands and looks at the exquisitely-appointed room with all its perfect arrangements, and says to herself—

> "Where every prospect pleases,
> And only woman is vile!"

What pride an English nurse would take in such a ward if only she had charge of it!

At six o'clock the two patients are still in labour, though the primipara's agony is nearly over. The midwife has put aside her needlework now, and is in attendance on the case, but she is irritated at the child's slow advent. "Push! push!" she cries to the woman in labour.

Mdlle. Anglaise ventures to suggest that inertia is sure to arise where no nourishment is given for nine hours; the midwife looks surprised.

After the baby is born the placenta cannot be expressed; but still no drink of warm milk is given the patient, and it is only after twice asking that she is supplied with a little cold water to moisten her parched lips. Wearily Mdlle. Anglaise wanders away, disheartened and sore at the lack of sympathy and lack of common-sense shown by her own sex. And yet they train their midwives for two years in Paris.

### Joseph.

The delicious whiteness of the Salle Blache is broken only by Joseph's blue jacket. He is standing up on his bed and doing a tottering dance that threatens to land him on the floor every moment.

"Joséf! Joséf!" calls the nurse, and she runs down the ward and picks him up and cuddles him and scolds him: Joseph crows. You cannot expect Joseph to keep still, for he is perfectly well. It is only because he has nowhere to go, and because all the nurses and the *surveillante* love him, that he is kept on here. Woe betide the doctor who would suggest discharging Joseph! How Mdlle. la Surveillante would gaze at him.

But just now the nurses are too busy to play with Joseph. This long ward has to be swept and then washed over with a cloth tied round the broom. It nearly breaks the nurses' hearts to wash their polished floors, but the stern edict has gone forth that polishing the floors is not so cleanly as washing them; and so dull, ugly floors break the otherwise perfectness of the Salle Blache. Failing his own particular nurses, Joseph (whose language is confined to croons, and who therefore doesn't mind bad French) condescends to accept the homage of the stranger and to ride about the ward on her back. First a little fellow recovering from pneumonia offers Joseph a sponge cake. Joseph is bidden to "embrace" the lad as thanks for the gift, but instead he hits the lad with his tiny hand. This is one of Joseph's jokes, and it never fails to make the ward ring with laughter.

Then Joseph is taken to one end of the ward, and from the top of a small flight of stairs looks down at the babies' ante-room. Really Joseph ought to be in the babies' room himself, but then he never is where he ought to be, and he prefers to be near Mdlle.'s table in the centre of the main ward. One of the babies is "Jean Jacques Rousseau," and, poor little chap, he is quite blind; but now, as he hears Joseph's croons above him, he breaks out in a feeble little voice into the first lines of the Marseillaise. Nearly all the children are sleeping under muslin curtains, for the flies are a terrible nuisance. A hot, kindly young nurse, from whose face the perspiration is streaming, is bathing one baby after another in cold water to try and get down some of the temperatures. The love of little children seems strongly mplanted in these good-natured women.

The stranger objects to taking Joseph to the top of the ward because she has not yet got over her English prejudice that whooping cough is infectious—and there are some very bad cases of whooping cough up there. Well, well! we used to nurse diphtheria in the general wards at The London at one time, and declare it never spread.

But now Mdlle. comes back from her lunch—Mdlle. with her charming manners, pretty face, and gentle voice—and Joseph will have no more of the stranger. The nurses group round Mdlle., and laugh at Joseph's struggles to escape to her; the little patients sit up and laugh also. What a cheery white ward it is! And how nice for the children not to be always being told to lie still and have their quilts put straight. In the Salle Blache they think more of the comfort of the child than of the straightness of the counterpane.

### The Infants.

There are two rows of them along two sides of the big white room. They are lying on long strips of cotton-wool laid on the floor, and their little red faces above their mummified bodies make quite brilliant patches of colour.

To the left two nurses sit surrounded with garments and dressings and bowls; each is at work on a baby's toilet. As soon as she has finished one child she pops him back in his place on the floor and picks up the next pink infant. The toilet only takes about five minutes, for the elaborate English bath has been discarded under the latest Parisian methods.

The child is swiftly wiped over with a bit of cotton-wool dipped in spirit and water, then it is mopped, and then powdered. Then its little chemise and blue bodice goes on, and round its waist is pinned a little square blanket, turned up over the toes, and then the little mummy is finished. There is no cord to dress, for it is cut off as close as possible, and then nipped with a pair of artery forceps, which are worn (wrapped up in wool) for the first few days of existence. This seems a very cleanly plan, but a small clamp might be invented to replace the forceps. Neither infants nor mothers are allowed binders. The babies certainly show less inclination to redness and soreness with the absence of washing, and risks of chill must be greatly decreased.

The pleasant young midwife who occasionally looks in to see that all is going well has pinned a bit of turquoise blue velvet round her neck to relieve the ugliness of her holland blouse. She has the most beautifully-kept and deft little hands, and gets through the hardest work with a daintiness that is truly Parisian. She is busy now doing the dressings for the mothers, while the nurses attend to the babies. But the infants are all finished, and one by one are picked up and carried back to the ward. Each wears a bracelet of tape on which its name is written, so that the children cannot be "mixed up" in the way that is dear to the novelist's heart. When a baby leaves the hospital the Assistance Publique presents it with two complete sets of clothing, consisting of two blue caps, two blue bodices, two chemises, two diapers, two small blankets, and two tiny white fichus. So thereafter it is the mother's fault if the cleanliness and simplicity of the hospital are no longer observed.

### The Victoria Commemoration Club for Nurses.

This club, located in The Hospital Building, 28 and 29, Southampton Street, was established nearly two years ago for trained nurses and associated workers. We are glad to say it grows apace both in numbers and popularity. There are already between 300 and 400 members. We hear nothing but nice things said as to its comfort, convenience, and management. Nurses who are beginning to understand the use of a club appreciate the homeliness and independence it secures to them. Town members make it their headquarters where they can drop in for half an hour to read, write, or refresh themselves. Suburban and country members can do the same, and have besides a resting-place for parcels to be collected later, a great convenience when shopping. Not very long ago bedrooms were added which country members coming to London for business appointments or for recreation find most useful. There are few clubs or other institutions, public or private, where the means and wishes of those patronising them are so studied. Meals are served at all times convenient to nurses, and the simplest fare to an excellent *table d'hôte* dinner can be procured. The dainty service and excellent cooking is a great attraction. All the advantages of this charming club can now be tested by any nurse who likes to join for the remaining quarter of the year—on October 1st, for the small sum of 5s., and the entrance fee of £1 1s.

### Autumn Novelties.

The rapid transition from almost tropical heat to colder days reminds us that autumn is upon us, and that warmer garments will be required to replace the lighter clothing of the warmest September on record. In the shop windows already are tempting displays of fashions for the coming winter, and those among us who are wise will begin to make provision, lest we be taken unawares. A visit to

MESSRS. GARROULDS, of Edgware Road fame, the other day, convinced us still further, if that were necessary, of the excellence and completeness of all their arrangements for the convenience of the nursing world. No detail seems to have been overlooked, and the choice and variety is endless. Their new catalogue contains several fresh designs both charming and original, and will be of great assistance to any nurse at a distance who is unable to make a personal selection. We admired particularly a cap of the becoming Marie Stuart shape, edged with either Valenciennes lace or spotted net quilling, which becomes flat for washing. Its name is the "Abbeville," and it is so pretty that we venture to prophesy for it a large sale. There are also some pleasing novelties in the way of strings, an especially dainty kind being made in hemstitched white washing silk. Those in cambric edged with lace are likewise attractive and fresh looking. An apron, the "Irena," struck us as being one of the best cut models we have seen. It is high to the throat and crosses at the back, where it fastens at the waist. It is made in two sizes at 2s. 6d. For those nurses who wear sleeves we noticed a very neat close-fitting kind made in either linen or cambric at 1s. 3d. per pair, which, being shaped to the arm, look very smart and workmanlike. The "Canterbury" cycling cloak will be a boon to district and country nurses. It is well cut and thoroughly waterproof, and is made in both light and heavy cloth to suit the taste of the wearer. For night nurses the "Chelsea" shoe has special attractions. It is made of warm black felt lined with scarlet flannel, and has the advantage of a double sole, which makes it twice as durable and prevents the foot from coming in too close contact with the ground. It is quite noiseless and very soft, and only cost 3s. 3d. In dress materials we admired the soft pretty shades of the "Halifaxes" and "Zephyrs." The former is everlasting wear and suitable for all seasons of the year. "Garrouldetta" is the name of another useful and charming fabric. For those who desire to make their own aprons nothing is more suitable than the "Tikord" linen. It is fine, strong, and has a beautiful smooth finish. Messrs. Garroulds have recently made several valuable additions to their "nursing requisites" department, the "specialite" chatelaine and the "Eva" wallet being a great improvement over existing designs. The "nurses' bag" is another delightful novelty, and happy is the possessor of such a treasure. Space forbids a further description of all that is to be seen and had at this wonderful emporium; our readers must pay a visit for themselves, and before leaving make a purchase of one of the fascinating little red leather pincushions that are to be had at a trifling cost in all shapes and sizes.

### MESSRS. DEBENHAM AND FREEBODY.
(Wigmore Street, W.)

Looking in for a few moments at this deservedly popular establishment we found some charming cloaks and bonnets on view. The bonnets become, if possible, more fascinating each season, and we quite lost our hearts to an especially attractive model called "the Dorothy." In cloaks, which for elegance of fit and style this firm stands unrivalled, we may mention the "Nora" and the "Dora" as two of the most becoming, and the "Victoria," which will always hold its own as a perfect cycling costume. We noticed one or two pretty caps and aprons, the latter being particularly well cut and finished off. Nurses' furniture is another specialite of this firm, and it is excellent, both in quality and appearance.

### HARROD'S STORES (LIMITED).
(Brompton, S.W.)

Here in this vast establishment our readers may revel to their hearts' content. Without exaggeration, its arrangements are so wide and complete that it is possible to go in at one door and come out at another armed *cap à pie* with all that the heart of woman can desire. Convenient as this undoubtedly is to the world at large, it is doubly so to nurses, whose time, as a rule, is very limited. Arrived at the linendrapery department, we find a vast assortment of dress materials in all shades and kinds. Some of the designs are very artistic and original, and will on that account be largely patronised. A useful nurses' cloak, by name the "Mildred," has much to recommend it, and can be had in black, navy blue, or grey for summer wear at 21s., and in a heavier make for winter at 25s. 6d. We admired a pretty, yet simple, cap, consisting of a plain band of muslin across the front edged top and bottom with Coventry frilling, and a crown drawn into position by a string at the back, as one likely to become popular. There are also several varieties of hemstitched collars and cuffs which have a very dainty appearance. In the boot and shoe department we were considerably struck by the comfortable and elegant appearance of a pair of shoes let in over the fronts with gussets of elastic. No laces or buttons to get out of order; a weighty consideration this for a nurse. Messrs. Harrod have also a good assortment of hygienic shoes of all descriptions, which alone are worth a visit.

### D. H. EVANS AND CO.
(Oxford Street, W.)

This enterprising firm is prepared to welcome nurses among its numerous patrons, and, with that end in view, keeps in stock an assortment of caps both cheap and becoming, and aprons of good design in the best materials. We admired a very good selection of cuffs and collars, some of the narrow hem-stitched linen varieties being elegant in the extreme. The "Bentick Shoe," designed for ward use, is a serviceable, comfortable-looking article, and deserves to be widely patronised.

### MESSRS. EGERTON BURNETT AND CO.
(Wellington, Somerset.)

The time-honoured Wellington firm is, as usual, in the van of fashion with its tempting array of flannels, serges, homespuns, winceys, and what not. It is almost bewildering to open the pattern box, which each season increases in bulk, and find oneself among hundreds of designs, each one more useful and beautiful than the other. Of course, for serges this firm will hold its own against all competitors, and as we look through bundle after bundle of that so appropriately described as the "Royal Serge" we feel it would be impossible to improve on it. Ever mindful of the needs of all their customers, we are glad to see the Messrs. Egerton Burnetts cater especially for nurses. Some beautifully fine serge cloth in navy blue, black, and grey is recommended as suitable for cloaks. It is a great convenience, and one which our readers will doubtless appreciate, to know that an excellent tailoring department will make up a garment on receipt of measurements. There are some particularly nice designs in rep cloth, and also some delightful little checked tweeds in various shades called the "Rutherglen." The "Severn," a useful woollen material at 1s. 6d. per yard, would make up into uniform, cycling, or other dresses, according to the shade selected. A special cycle serge is quoted at 2s. 11½d. per yard of 54 inches, and has a very useful, sensible appearance. There are some beautiful white serges, shrunk for dresses. and also an elegant white wincey, which is ideal summer wear. These goods deserve special mention, as they are really admirable of their kind, and it is sometimes difficult to know exactly where to get a suitable white material for boating purposes. Flannels there are in endless variety and of excellent quality, besides other materials too numerous to mention. Our readers could not do better than satisfy themselves of the accuracy of our representations by sending without delay for a box of patterns.

### A NEW FEEDING CUP.

We have received from Mr. C. B. Hoult, 4, St. Mary Axe, London, E.C., a specimen of a new "Antiseptic Feeding Cup," the invention of a nurse. In shape and form this latest feeder is similar to those with which we are all familiar, its special feature being a movable spout or nozzle, made either of nickel, aluminium, or silver, which admits of a more thorough cleansing than can be attained in the case of the ordinary article. The cup has decided advantages to offer: risk of breakage is considerably diminished, the spout being that part of an earthenware or china feeder which most frequently comes to grief; and when the metal spout is removed every part of the vessel can be seen and easily cleansed, the short neck of the cup having a large aperture for this purpose. The spouts are made interchangeable by a uniform gauge of size. The one objection which will occur to those who have put the cup into practical use is that, though admirable for cold or slightly warmed liquids, no really hot food can be put into it without the danger of the metal spout scalding the patient's lips. Metal is, of course, a better conductor of heat than china ware, and a spout made of that material will get unpleasantly warm if the temperature of the contents be at all high. From every other point of view Nurse Lewis's Feeding Cup is highly to be commended, and will doubtless become widely appreciated. The price of the feeders made in white ware, with German silver spouts, is 15s. per dozen; more elaborately decorated ones, with hall-marked silver spouts, may also be had, and the prices of these can be obtained upon application to Mr. Hoult.

### The London School of Medicine for Women.

### OPENING OF THE WINTER SESSION.

At the opening of the winter session of the London School of Medicine for Women, at the Royal Free Hospital, on Tuesday evening, October 4th, the introductory address was delivered by Dr. J. Walter Carr, senior assistant physician to the hospital. Dr. Carr treated his hearers to very excellent advice full of sound common sense. Choosing as his subject, "The Influence of Fashion on Medicine," Dr. Carr briefly reviewed the various methods of treating fevers which had found more or less temporary favour during the progress of the present century, from the excessive use of venesection, the cuppings and blistering, and lowering treatment in vogue in its early years, to the adoption of the opposite extreme, when enormous quantities of alcohol were freely administered to fever patients, and thence to the reaction of which the establishment of temperance hospitals might be regarded as the outward and visible sign. Then came the fashion of reducing temperature by the outward application of cold, or by powerful drugs, such as antipyrin, while at present animal extracts and anti-toxins were in the ascendant—all methods useful in their places, if not pushed into excess and so brought into discredit. The lesson to be learnt was the need for the cultivation of a more judicial spirit, a greater steadfastness; not adopting some new drug or method one year and entirely discarding it the next, but seeking the good in every system of treatment, avoiding on the one hand the danger of pushing it to excess, and on the other of ignoring it altogether. It was to be feared that there was a danger of overdoing the use of specifics, and while rejoing in the powerful effect of such drugs as salicylate of soda in rheumatism, or colchicum in gout, it was to be remembered that the ideal in medicine must ever be to discover the causes of disease, and by removing them prevent the recurrence of the disorder, as had been successfully done in the case of typhus, rather than find specifics which would act in merely temporary alleviation. The most successful physician was he who could influence individual men and women to heed the laws of health. Dr. Carr went on to speak of the revolution which had taken place in nursing during the present century, of the danger of a reaction, and of the fear that, with the enormous increase in the number of trained nurses, there would come a falling off in quality. This was more the case with the private nurses than with those who worked amid the greater restrictions of hospital life, but it was with fear and trembling now that a medical man sent for an unknown private nurse. Dr. Carr was sarcastic at the expense of those nurses who "thought more highly of themselves than they ought to think," and expressed a fear lest it should become the fashion amongst the middle classes to leave the nursing of the sick entirely in the hands of strangers, not merely in such cases as typhoid or pneumonia, which demanded skilled nursing, but in minor ailments. Dr. Carr concluded his lecture by considering the present position of medical women, asking, Were they destined to take a permanent place in medicine, or was the movement to be a mere passing fashion? The question might seem superfluous in face of the rapid progress now being made; but the perils of prosperity were sometimes more to be feared than those of adversity, and if the ease with which women could now enter the profession led to the admission of those who were unsuitable a reaction might follow which would seriously retard the movement. He therefore urged upon the students to consider the responsibility which rested upon each one of them.

Mr. and Mrs. Burt, Mrs. Garrett Anderson, and Mrs. Thorne were amongst those present on the platform. After the address prizes were distributed to the successful students by Mrs. Burt.

### The St. Albans' Diocesan Nursing Institution.

A PUBLIC meeting in aid of the St. Albans' Diocesan Nursing Institution and the Claughton Convalescent Home was held recently at Colchester. The following resolution was passed: "That this meeting hears with much satisfaction the report of the general working of the nursing institution, and also that the Claughton Home having proved so successful the meeting pledges itself to assist in defraying the remaining debt of £136."

### The Infant Life Protection Act.

THE appointment of a woman to fill one of the posts opened by the new Act seems to be very suitable, and it is to be hoped that this course will be generally adopted. Mrs. S. Lofthouse has been appointed inspector of the Kingston Union. She is the widow of a physician. The salary is £100 per annum. Mrs. Loftus competed against 177 candidates, amongst whom 31 were women.

### A Book and its Story.

THE HOUSE OF HIDDEN TREASURE.*

MAXWELL GRAY'S "House of Hidden Treasure" is a romance, without any particular plot; quite un-up-to-date, and entirely simple and pleasant in its details.

Early in the far-away days of crinolines, pork-pie hats, and Piccadilly weepers, Caroline Hardwin, the idolised daughter of an autocratic father, made a runaway match with a young dragoon officer, Clarence Dorrien. Sir Geoffrey Hardwin, smarting deeply under the loss of his daughter, finds the solitude of Hardwin Hall insupportable, so he invites to Hardwin a nephew of his late wife's, Brinson Hythe, whom he adopts as his heir in place of Caroline. Hythe, who takes the name of Harbord, after a family estate, is a rejected suitor of the disinherited Caroline. Later on he becomes known to the Dorrien family by the fitting title of "Judas Brimstone," bestowed upon him by Caroline's clear-sighted daughter Grace, known vicariously as "Grace," "Disgrace," or "Jack." It is around Grace, as heroine, that the interest of the story centres. An escapade of Grace's, when they were quartered at a well known military and naval port, serves to illustrate the irrepressible daring of the girl.

One evening, after a not unusual passage of arms with her mother, she locks herself in her room, and escapes by the window into the cool dusk of the summer night.

Soon the silence of the night is broken by the sound of a lovely mezzo-soprano voice, accompanied by a guitar, singing the gay, light-hearted, yet passionate songs of Spain. The intervals are filled with dancing, and this handsome young woman, in the dress of a Spanish peasant, draws an ever-increasing crowd of admiring spectators.

"Such a dance! The joy of full, fresh life, the lightness of hovering breezes, tumbling wave crests, wind-tossed autumn leaves, the incessant motion of a ball tossed and caught by a leaping fountain; grace of raised and circling arms, smiling lips, and quick snap of castanets. Then slower movements, deeper song, silent castanets, drooping arms, . . ." "and the beautiful musician glided, almost melted, away through the crowd towards the dark, sighing sea."

"Who could the singer be?" No mere street musician; possibly some operatic star making a holiday, and perhaps winning a wager. "Not an opera singer," Mrs. Dorrien thought, "the voice was not sufficiently trained." So with a density of ear and vision, peculiar in fiction to similar situations, the heroine goes unrecognised by her nearest relatives. Since the Spanish incident Grace had been ominously, almost pathetically "good." Something was brewing in her active brain, of course, and as usual in family difficulties, it is she who acts with decision. Her father is off to Homburg or Baden, *en garcon.* She seeks him out, has a short and startling interview unknown to her mother, unfolds her plan to him of starting off and paying a "surprise" visit to her grandfather, Sir Geoffrey, relying upon her charms to take the old man's heart by storm, and gain forgiveness for her mother.

So Grace starts in the care of the faithful Mursell—an admirably depicted character—and on the evening of their arrival, oblivious of the fatigue of a long journey, she hurries forth into the moonlight night for a ramble in the park. "A bend in the white road brought them in sudden sight of the fairy palace, all ablaze beneath the pale moonlit sky, and looking as if piled there on its commanding eminence by enchantment."

The next day Grace lays siege to the citadel and is repulsed. Scant courtesy is shown to her by Hythe Brinson and Sir Geoffrey's servants, who, acting under Brinson's direction, refuse to admit her.

She makes her second attack on a public day, when a portion of the house and grounds are thrown open. Unattended, she comes suddenly upon her grandfather soliloquising in an adjacent arcade.

"'Yes, very lonely; a worn and weary pilgrim; a poor forsaken old man. The wife of his youth down there in the churchyard; his only child lost and impenitent." With characteristic *savoir faire* Grace makes herself mistress of the situation. After a somewhat stormy interview Sir Geoffrey succumbs, and the same evening sees her installed with Mursell in a suite of rooms at Hardwin, partaking of her grandfather's lavish hospitality.

"Judas Brimstone," finding himself outwitted, accepts the situation, and joins apparently in the adoration and devotion of Sir Geoffrey to their charming guest. But one day when riding a valuable horse, her grandfather's latest gift, Grace was unseated by a stray shot from a gun; her horse was shot under her, and she escaped with a severe shaking. Grace joins her people again; they go to Mentone, where she meets her fate in the son of an old brother officer of Colonel Dorrien's, invalided from Lucknow, and bearing traces of its horrors in a wasted frame and the mark of a Tulwar scar visible on occasions.

All the strong vitality of Grace's youth, all the latent sympathy and sweetness of a generous, passionate nature, is moved by his forlorn appearance. And when days of happy convalescence set in, he returns her kind offices by lessons in Italian and readings from Dante and Tasso, "in a garden looking on olives and carobs, aloes and lemon groves, peach and mimosa bloom." So the two find Eden again. But as this is a story of frustrated happiness, deferred hopes, and much misunderstanding, these two part—he to return to his regiment, never knowing or realising her affection, from a Quixotic, mistaken feeling on Grace's part that, as it is her sister whom he loves, she must hide her feelings from him. Years pass on; Colonel Dorrien dies. To Grace falls the charge of her mother, prematurely broken down by a life of anxiety, and her heroism is called into full play in the crisis following Colonel Dorrien's death. Into the shadows and silence of the old house she passes with her mother.

In the prologue we first meet her here, leading her quiet, devoted life of self-sacrifice, beautiful still in spite of having passed her first youth. Brinson Harbord dies, and she finds herself sole heiress of Sir Geoffrey's property. Not wishing to leave the old house, she lives on there surrounded by loving friends.

Her old lover, Captain (now Lord) Hilton, had married, and lost his wife. After years of separation and misunderstanding they are to meet again once more. But Grace is fading imperceptibly. She is buoyed up with blissful anticipations of seeing the face of her hero and life's one love. "She lay still in unutterable peace all day long, singing in her heart a perpetual joyous refrain, 'He is coming; he is coming; at last! at last! at last!'"

A quick, firm step through the hall and on the uncarpeted stairs; one moment more, and Mark Hilton, "tall, soldierly, with grey hair, spare frame, level glance, and commanding presence, stands in the presence of his beloved. . . . He saw the outstretched hands, the unutterable love of a life in the rapidly paling face . . . but though he paused no moment, though his step was rapid across the room, he was but just in time to receive the last happy sigh of the passing soul." So the lovers meet, and part—she to where "Beyond these voices there is peace," he to face life alone once more. And it is with regret that we leave them thus; but the author's and the reader's mind are not necessarily identical in points of view. An old-fashioned story one feels should have a happy ending, and "The House of Hidden Treasure," as we have before remarked, is quite out-of-date enough, and has sufficiently the charm of a past-day novel to lead us to expect this desirable conclusion.

* By Maxwell Gray. (Heinemann, London. 6s.)

### Hospital Secrets.

As Told by the Steward.

### I.—"SANDY."

Everybody is fond of Sandy. He is a big, kind-hearted simple-minded fellow of thirty-seven. He stands six feet two inches in his stockinged feet. As a Grenadier Guardsman he saw much of life, and as a hospital porter he has added considerably to his experience. He has been with us nearly five years, and now, unless for some very good reason, we who are in authority would not be without him. At this, the Royal Central West Hospital, we have almost a score of hard-worked porters, and Sandy, although not the senior amongst them, is the hot favourite. He has such a way with him. The women folk—from the all-powerful matron down to the last-engaged little scullerymaid—have gradually come to look upon him as an indulgent brother. If one of the half-hundred nurses is going away for a brief holiday and wants someone to see her box safely placed in the care of the apathetic cabman, it is Sandy who comes upon the scene just at the right moment, and who says and does all that is required to put the departing one at ease. If there is some trifling commission that can best be done by one of the men in his own time, that man is certain to be Sandy.

His name is not Sandy. Pet names need not be at all close in resemblance to the name given at baptism. Sandy's name, as becomes a true-born Scotsman, is Robert James Alexander Campbell. His mother, who still flourishes in her sweet little native village away in the farthest north of bonnie Scotland, is a woman who comes of a large family; and when Sandy was born it pleased her to perpetuate the first names of three of her brothers in her wee laddie. Sandy had some six or eight years' schooling, and then went out as signal boy on a small local line that ran by the foot of the garden that was tended by his father when his day's work on the railway was done. But at eighteen, when Sandy was as tall as his father, and a great deal stronger and handsomer, he left his home and took the Queen's shilling. At the end of fourteen years his colonel and the other officers, on learning that he was returning to the life of a civilian, gave him excellent testimonials, and secured him a post under me at the Royal Central West Hospital, London.

And now he has been with us nearly five years. He has built up for himself a reputation that a Bishop might envy. His eye is clear, his back is straight as 'twas in his early drill days, his hand is steady, and his heart is of the best. He has married since he came to us. It is four years since he wooed and won Martha Simpson, a pleasant-faced little Londoner of respectable parentage and honest thoughts. They have a little boy of three who is named James, but who is known to all and sundry as Jemmie. Chubby-faced and chubby-limbed Jemmie, who does not leave his cot at his parents' bedside in the morning until Sandy has been at work for two hours, and who goes to his cot night by night —Sundays excepted—exactly an hour after Sandy's return home. On three Sundays out of five Jemmie has his dad for the whole day, for Sunday duty at the Royal Central West is unavoidable. And how happy Jemmie makes Sandy, and Sandy makes Jemmie, on those days when they are together.

Until a few months ago Martha and the boy lived with Sandy near to the hospital. They had three rooms in some tall buildings that stand by the side of a busy thoroughfare in Clerkenwell. Now, however, they live eight miles away in one of a long, long row of tidy little houses, the windows of which overlook a far-stretching green field. The facilities offered to workmen by one of the large railway companies has made this easy of accomplishment.

Jemmie loves the country. And Martha can let him go out and play, whilst she makes progress with the housework, without feeling that he is in danger of being run over, or stolen, or otherwise entrapped by the wiles of wicked townsmen. Jemmie is happy the live-long day, and so is Martha, and so is Sandy. Sandy makes Martha happy; Martha makes Jemmie happy; and, boylike, Jemmie retaliates and makes them both happy.

Last summer, when Sandy had ten days' holiday granted him, all the three of them went by a cheap boat excursion from London Bridge to Scotland. Martha prevailed upon Sandy to take her and the boy to see the very high hills that lie about and shelter his native place. The trip meant economy for months, yet it was brought about. And Jemmie had a new knickerbocker suit to go in too! Perhaps Martha knew more about the economy necessitated by the outing than either Jemmie or Sandy, for she used to say that she did not care much for meat, and that her winter boots were so easy that she preferred them to new ones for walking in.

They reached the cottage of Sandy's dear old mother safely, and they saw the hills and the heather, and all sorts of bits of nature that previously had been as nothing to them. Jemmie's amazement was not nearly as deep as Martha's, as was not unnatural, but both of them laid hold of Sandy for support when first viewing the hills, up which Martha rightly expressed herself when she hinted that no London tram-car could make ascent. And how Jemmie enjoyed the fresh eggs laid by Granny's fowls, and the new milk, purchased from the neighbouring farmer, while still warm. And how he cried when Granny kissed him good-bye at the side of the steamer, when leaving for home.

Months have passed since then, and Sandy and Martha have had their first real quarrel. It happened thus. Two old friends of Sandy's, two men whom he knew and played with as boys, came on a visit to London. They were aware that he worked at the Royal Central West, and they called here as he was one evening about to change his uniform for his own clothes before starting for home. The two friends had much to tell him and much to ask him, but the time at their disposal precluded a journey with him to Martha's fireside; what then more likely as a suitable way out of the difficulty than a call at a public-house and a chat in the gaudily-lit bar.

Sandy was never a man for much ale. Two glasses rendered him argumentative, and three made him obstinate.

He was three hours late on arrival at home, and Martha was a little impatient. She was not accustomed to such treatment, and she rightly enough sought for an explanation. Sandy had had four glasses, and was incapable of any explanation. He awoke next morning feeling far from well, and Martha's unusually quiet and distant manner made him feel still worse. Had he been a man given to outbursts of temper matters would undoubtedly have reached a crisis, but temper and Sandy were strangers, and he left the little home in silence, and without the bestowal of the kiss that Martha was in the habit of receiving morning by morning on the back door-mat as she opened the door for him.

All day long Sandy worked badly. He did his routine duties mechanically, and his duties requiring thought abominably. But it was thought he was unwell, and he escaped serious reprimand. When night came he left at the usual time and made his way to the station, where he parted from two fellow-porters who bade him "so-long."

Left alone, he fumbled about with the return half of his railway ticket for some minutes and then arrived at a sudden decision, regained the street, and crept stealthily back to the hospital. Merely by chance his entrance was unobserved, and he made his way quickly to a distant room in the basement and threw himself down upon some lumber and sobbed as a child. He and Martha were not good friends, and the fact was more than he could bear. All the day through he had half expected her to come up to town and call at the hospital and give him the kiss of forgiveness, but she had not done so, and now he felt that return to her and Jemmie that night was impossible.

Had anyone in the establishment known of his secret sorrow and his whereabouts, how gladly would half the arms in the buildings have been linked in his, and the trouble smoothed away. But there, not a single soul knew of it.

He tossed about in the damp and the darkness all the weary hours of darkness through, and at five in the morning the night-watchman heard sounds, and discovered him. I, as the steward, was informed, and, as the one responsible to the Committee of Management for all that goes wrong concerning the porters, did what I could to comfort him. Anger played no part in my little speech of remonstrance, and when daylight arrived, and the train service resumed its sway, I accompanied him down to his home, where Martha, with red and swollen eyes, and with Jemmie hugged to her breast, anxiously awaited him.

Sandy has had a bitter lesson. His big simple heart has had the pain of a cold thrust from without, and wants no more.

Nobody at the Royal Central West but myself knows anything about the passing trouble. Perhaps one or two of the more observant women-folk thought something was amiss during the day of Sandy's unwonted quietude, followed by the day's absence, but of proof there is none.

I feel somewhat mean in making "copy" of Sandy's misfortune, but my conscience bounds up again when I recall that as Sandy brings me my Hospital every Thursday, the look in his face correctly read may be interpreted: "I dinna ken how he can read the likes o' them unintereestin' papers."

### Pictures from Florence.

By a Correspondent.

### THE FLORENTINE HOSPITAL.

There is a pretty story told about the founding of the large general hospital which receives the sick poor of Florence and its neighbourhood. According to tradition, Folco Portinari, the father of Danté's Beatrice, had an old servant who devoted herself to helping the sick and feeble. With her master's permission she received them into the Portinari Palace, and gave up her whole time to them. At last there were so many invalids brought in to be cared for that there was little room for anyone else. Then Folco, who was a kind and generous man, began to think that the sick should have a building of their own—one specially adapted to their needs and furnished with all the conveniences of the thirteenth century. He erected his hospital close to the site of the present building, which was built when the first one became too small for the number of sick who crowded to its doors.

We went to see the hospital on a burning July day, when the thermometer stood 94 in the shade. It was noon when we reached the door, and there were a number of people sitting under the portico waiting for the clock to strike the hour. They were friends of the patients, who, as usual in Italian hospitals, were admitted to the wards between twelve and one. The men sat leaning against the wall half asleep, but the women were chattering at the top of their voices and keeping up a continual movement with their small paper fans. Many of them had baskets or small bundles which they hoped to get through the doorway without too much inspection.

We all went in together when the clock sounded. A regular wave of footsteps and voices overflowed into the hall and along the passages. The sleepy porter made an attempt to overhaul a few of the baskets, but most of them got past without any searching whatever.

The big wards, with their whitewashed bare walls and red-tiled floors, looked cool and pleasant after the blazing heat outside. Their very emptiness and want of ornament, which makes them look so cold and unhomelike in the winter, were an advantage now. The bed linen was hand-woven, pleasant to the touch, though naturally of coarse quality. The patients looked very comfortable and much cooler than their perspiring visitors. All the women-patients and many of the men had their small paper fans, which they kept using all the time.

There were nuns in the Franciscan uniform in each of the wards. They were rather busily engaged in keeping an eye on the baskets and bundles. Still, to the mind of an English nurse there was too little interference with the indigestible articles of food that found their way to the patients. We kept on looking out for a sister of whom we might ask a few questions about the hospital, and found one at last in the kitchen, into which we got by taking a wrong turning. There were dozens of great copper pans all round her, and about six women engaged in polishing them under her directions. She had a kind, sensible face, and was ready to give us any information we wanted.

We had read in the guide book that the hospital contained about two thousand beds, and our hearts were sore and much humbled, for we had hitherto considered the London the largest hospital in Europe. The nun relieved our minds. There were about five hundred occupied, she said, and room for a good many more. But she shook her head at the suggestion of two thousand.

There are about fifty nuns in charge of the nursing. They belong to one of the orders of St. Francis, and wear heavy brown cloth uniforms. Their head-dress consists of a white linen cloth wound round head and chin and falling on the shoulders. It resembles those worn by St. Clara and St. Catherine in the pictures of the Siennese painters. When the nuns go out of doors they put on the top of the head-dress an enormous leghorn hat, called a monachina. These hats are so large that they almost hide the face. They give a good deal of trouble in a light wind, such as sweeps the streets of Florence in the spring. The infirmiere, or nurses, who work under the sisters do not wear uniform.

Coming out of the hospital we went in at a side door to see the curious monument of Monna Tessa, the old serving woman, who was the true founder of the hospital. Her master has a monument in the church close at hand. It is very pleasant to think of the comradeship in good works that existed between the Florentine noble and his old servant, and one is glad to know that they are not forgotten.

There is an exquisite picture by Fra Angelico in the nuns' dormitory, and the hospital possesses a small gallery. Its gem is a wonderful picture by Van der Goes.

### Wants and Workers.

[The attention of correspondents is directed to the fact that "Helps Sickness and to Health" (Scientific Press, 28 & 29, Southampton Stree Strand, London, W.C.) will enable them promptly to find the most suitable accommodation for difficult or special cases.]

Miss D'Avenport would be very glad to hear of anyone who could let her have a felt jacket for spinal curvature for a poor child. Address Cottage Hospital, Ludlow.

### For Reading to the Sick.

LOVING ALLEGIANCE.

**Verses.**

O MASTER, at Thy feet
I bow in rapture sweet!
Before me, as in darkling glass,
Some glorious outlines pass
Of love, and truth, and holiness, and power;
I own them Thine, O Christ, and bless Thee for this hour.

Often through the heart is pealing
Many another voice than Thine,
Many an unwilled echo stealing
From the walls of this Thy shrine;
Let Thy longed-for accents fall;
Master, speak, and silence all. —*F. R. H.*

Hearken, hearken!
God speaketh to thy soul,
Saying, "Oh, thou that movest
With feeble steps across this earth of Mine
To break beside the fount thy golden bowl
And spill its purple wine:
Look up to heaven! and see how, like a scroll,
My right hand hath thine immortality
In an eternal grasping! thou that lovest
The songful birds and grasses under foot
And also what change mars and tombs pollute—
I am the end of love! Give love to Me."
—*E. B. Browning.*

Oh, work Thy works in God! He can rejoice in nought
Save only in Himself and what His self has wrought.
—*Trench.*

All common things, each day's events,
That with the hour begin and end,
Our pleasures and our discontents,
Are rounds by which we may ascend.

**Reading.**

"MASTER!"

"I have called Thee by Thy name" (Is. xliii.) That was quite enough. The powerful sunshine of His love was focussed into that white beam of sevenfold light, and the whole soul was concentrated into the responsive love-flash, "Master!"

When that word has truly gone up from the soul to Christ, then we have felt what we can never put into any other words. It is the single demand of soul expression, and we have cast it at His feet for ever. He accepts it; for how wonderfully sweetly falls His direct answer, "Ye call Me Master and Lord: and ye say well; for so I am." Think of this seal of approval being set upon the name we so love to give Him. "Ye say well." . . . And yet we only uttered the one word, "Master!" The true utterance of it is the very oath of allegiance. We cannot, must not, dare not, will not henceforth serve "two masters," nor the still more subtle "many masters." The word has been breathed into His heart, and He will treasure it there, and keep it for us. We have found One whom we can trust implicitly and rest upon entirely. We have put our lives into His hand. We have burned the bridge behind us, because we are quite sure He is the Captain of our salvation. We have entered His service for ever. We have given our allegiance unreservedly because we confide in Him unreservedly. There is no question about it. "I know whom I have believed," and therefore I say, "Master!"—*F. H.*

### Where to Go.

THE QUEEN'S JUBILEE NURSES' INSTITUTE.—A matinée performance will be held at the Empire Theatre on Thursday, November 10th, in aid of the institute.

### Notes and Queries.

**The contents of the Editor's Letter-box have now reached such unwieldy proportions that it has become necessary to establish a hard and fast rule regarding Answers to Correspondents. In future, all questions requiring replies will continue to be answered in this column without any fee. If an answer is required by letter, a fee of half-a-crown must be enclosed with the note containing the enquiry. We are always pleased to help our numerous correspondents to the fullest extent, and we can trust them to sympathise in the overwhelming amount of writing which makes the new rules a necessity.**

**Every communication must be accompanied by the writer's name and address, otherwise it will receive no attention.**

*Superfluous Hairs.*

(12) Could you tell me of any safe and permanent remedy for the removal of superfluous hairs on the face, apart from electrolysis.—*M. A. H.*

You had best take a doctor's advice on this point.

*Massage.*

(13) Where are single lessons in massage given?—H.

Write to Mrs. Creighton-Hale, 89, Mortimer Street, Cavendish Square, W., or to The Trained Masseuses' Society, 12, Buckingham Street, Strand.

*Training.*

(14) Kindly give me your opinion as to the advisability of re-entering a hospital for a three years' certificate. I have had over six years' nursing experience.—*K. S.*

If you intend to continue district nursing there is no need for you to hold a three years' certificate. But if you wish to take up one of the higher posts in an institution you must certainly possess a three years' certificate.

*District Nursing.*

(15) Is it possible to become a district nurse without hospital training? I have had three years' experience in workhouse nursing, and hold a certificate of the British Lying-in Hospital for monthly nursing. —*Alice.*

A nurse trained in a recognised workhouse infirmary is quite eligible for a position as district nurse.

*Cottage Hospital Training.*

(16) Is the cottage hospital at Great Ormesby a good training school, and will a three years' certificate gained there be looked upon as a good recommendation for a nurse in private work afterwards? Can you say the number of beds there?—*W.*

Training at a cottage hospital, however good, is never reckoned equal to that at a larger general hospital. If you refer to North Ormesby, Middlesbrough, the Cottage Hospital contains 60 beds.

*Home for Epileptic Case.*

(17) Are there more homes than one where a girl about 18 can be sent who suffers from epileptic fits? How should an application for admission be made?

There is the Meath Home for Epileptics, Westbrook, Godalming, and there is also the Epileptic Colony at Chalfont St. Peters, Bucks, which is the home of the National Society for the Employment of Epileptics. Write to the secretary of each of these associations for particulars.

*Training.*

(18) Would two years' training at an incurable home of 30 beds and one year's training at a general hospital with training school entitle me to a three years' certificate?—*Nurse L.*

No. Neither institution could give you a three years' certificate under the circumstances.

*Accouchement Outfit.*

(19) Can you give me the address of a firm where I can obtain the guinea parcel of requisite articles needed at obstetric cases which I saw advertised in THE HOSPITAL some months ago?—*E. M. S.*

We conclude you refer to the guinea accouchement outfit (Hartmann's) supplied by the Sanitary Wood Wool Company (Limited), 26, Thavie's Inn, Holborn Circus, London, E.C. These are supplied to nurses at the reduced price of 19s.

*Nursing Lectures in Paris.*

(20) Can you inform me whether there are courses of lectures in Paris (in either French or English) on nursing or obstetrics?—*S. E. S.*

We are making inquiries, and hope to be able to give you some information next week.

*Weir-Mitchell System.*

(21) Kindly let me know if there is a hospital in London where they teach males the Weir-Mitchell system of massage?—*K.*

Write to Mrs. Creighton-Hale, 89, Mortimer Street, Cavendish Square, W.

*Wintering in India.*

(22) Can you tell me how to proceed to get to India for the winter with an invalid?—*Sister A.*

We know no other means than advertising. You might inform the Lady Superintendent of the Nurses' Co-operation, 8, New Cavendish Street, W., who might know of someone wishing to take a nurse to India.

**ANSWERS REQUESTED.**

(23) If at a country house, where could a nurse get a sheet of mackintosh disinfected and obtain sterilised towels for operation?—*B.*

**ANSWERS.**

*Disinfection of Bed-Pan.*

(24) Disinfection writes: Nurse Mary can easily disinfect the rubber bed-pan by washing and soaking it in "Carbolacene." "Walker," of Liverpool, is the manufacturer. I can recommend it with confidence for bandages or mackintosh sheeting, &c.

TAORMINA.

**A View of Mount Etna from the Greek Theatre.**

## Figures and Tables

**Figure f1:**